# 4-[(*E*)-2-Furylmethyl­eneamino]-3-phenyl-1*H*-1,2,4-triazole-5(4*H*)-thione

**DOI:** 10.1107/S1600536808021806

**Published:** 2008-07-19

**Authors:** Hoong-Kun Fun, Samuel Robinson Jebas, K. V. Sujith, P. S. Patil, B. Kalluraya, S. M. Dharmaprakash

**Affiliations:** a X-ray Crystallography Unit, School of Physics, Universiti Sains Malaysia, 11800 USM, Penang, Malaysia; b Department of Studies in Chemistry, Mangalore University, Mangalagangotri, Mangalore 574 199, India; c Department of Studies in Physics, Mangalore University, Mangalagangotri, Mangalore 574 199, India

## Abstract

In the title mol­ecule, C_13_H_10_N_4_OS, the triazole ring makes dihedral angles of 16.14 (9) and 58.51 (11)°, respectively, with the phenyl and furan rings. Intra­molecular C—H⋯N hydrogen bonds generate *S*(5) and *S*(6) ring motifs. In the crystal structure, centrosymmetrically related mol­ecules are linked *via* N—H⋯S hydrogen bonds to form dimeric pairs, which are inter­linked *via* C—H⋯O and C—H⋯π inter­actions.

## Related literature

For the biological activities of triazole derivatives, see: Clemons *et al.* (2004 ▶); Glerman *et al.* (1997 ▶); Holla *et al.* (2003 ▶); Johnston (2002 ▶); Kane *et al.* (1990 ▶); Kkgzel *et al.* (2004 ▶); Modzelewska & Kalabun (1999 ▶); Rollas *et al.* (1993 ▶); Shujuan *et al.* (2004 ▶); For bond-length data, see: Allen *et al.* (1987 ▶). For graph-set analysis of hydrogen bonding, see: Bernstein *et al.* (1995 ▶).
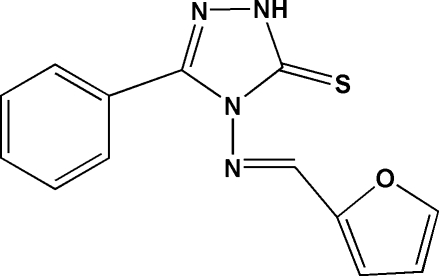


## Experimental

### 

#### Crystal data

C_13_H_10_N_4_OS
*M* *_r_* = 270.31
Orthorhombic, 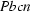

*a* = 27.4006 (6) Å
*b* = 11.4940 (3) Å
*c* = 7.7886 (2) Å
*V* = 2452.96 (10) Å^3^
*Z* = 8
Mo *K*α radiation
μ = 0.26 mm^−1^
*T* = 100.0 (1) K
0.40 × 0.13 × 0.10 mm

#### Data collection

Bruker SMART APEXII CCD area-detector diffractometer
Absorption correction: multi-scan (*SADABS*; Bruker, 2005 ▶) *T*_min_ = 0.829, *T*_max_ = 0.974
40042 measured reflections
3627 independent reflections
2573 reflections with *I* > 2σ(*I*)
*R*_int_ = 0.071

#### Refinement

*R*[*F* ^2^ > 2σ(*F* ^2^)] = 0.043
*wR*(*F* ^2^) = 0.125
*S* = 1.05
3627 reflections
176 parameters
1 restraint
H atoms treated by a mixture of independent and constrained refinement
Δρ_max_ = 0.26 e Å^−3^
Δρ_min_ = −0.32 e Å^−3^

### 

Data collection: *APEX2* (Bruker, 2005 ▶); cell refinement: *APEX2*; data reduction: *SAINT* (Bruker, 2005 ▶); program(s) used to solve structure: *SHELXTL* (Sheldrick, 2008 ▶); program(s) used to refine structure: *SHELXTL*; molecular graphics: *SHELXTL*; software used to prepare material for publication: *SHELXTL* and *PLATON* (Spek, 2003 ▶).

## Supplementary Material

Crystal structure: contains datablocks global, I. DOI: 10.1107/S1600536808021806/ci2631sup1.cif

Structure factors: contains datablocks I. DOI: 10.1107/S1600536808021806/ci2631Isup2.hkl

Additional supplementary materials:  crystallographic information; 3D view; checkCIF report

## Figures and Tables

**Table 1:** Hydrogen-bond geometry (Å, °) *Cg*1 is the centroid of the C3–C8 ring.

*D*—H⋯*A*	*D*—H	H⋯*A*	*D*⋯*A*	*D*—H⋯*A*
N1—H1N1⋯S1^i^	0.85 (2)	2.42 (2)	3.265 (2)	169 (2)
C4—H4*A*⋯N2	0.93	2.55	2.859 (2)	100
C6—H6*A*⋯O1^ii^	0.93	2.59	3.347 (2)	139
C8—H8*A*⋯N4	0.93	2.29	2.942 (2)	126
C5—H5*A*⋯*Cg*1^iii^	0.93	2.92	3.522 (2)	123

Symmetry codes: (i) 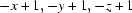; (ii) 
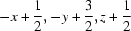
; (iii) 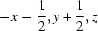.

**Fig. 1.**
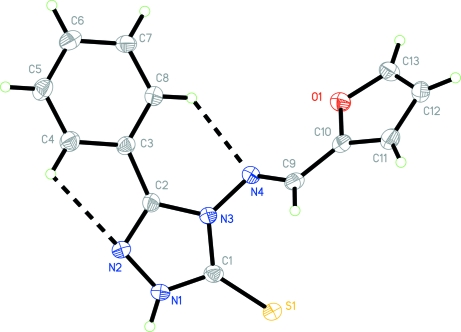
The molecular structure of the title compound, showing 50% probability displacement ellipsoids and the atom-numbering scheme.

**Fig. 2.**
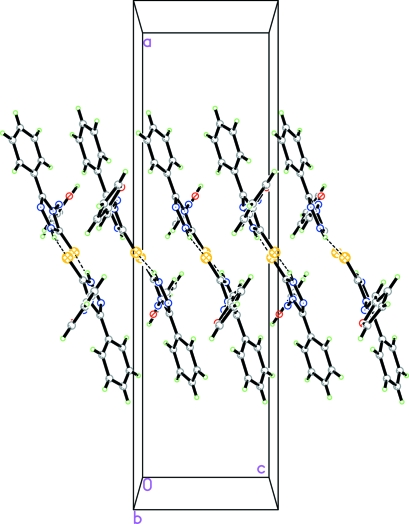
The crystal packing of the title compound, viewed along the b axis. Hydrogen bonds are shown as dashed lines.

## supplementary crystallographic information

### Comment

1,2,4-Triazoles and their derivatives are found to be associated with various biological activities such as anticonvulsant (Kane *et al.*, 1990; Kkgzel *et al.*, 2004), antifungal (Rollas *et al.*, 1993), anticancer (Holla *et al.*, 2003), anti-inflammatory (Modzelewska & Kalabun, 1999) and antibacterial properties (Glerman *et al.*, 1997). Several compounds containing 1,2,4-triazole rings are well known as drugs. For example, fluconazole is used as an antimicrobial drug (Shujuan *et al.*, 2004), while vorozole, letrozole and anastrozole are non-steroidal drugs used for the threatment of cancer (Clemons *et al.*, 2004) and loreclezole is used as an anticonvulsant (Johnston, 2002) drug. In view of the above properties, we have synthesized the title compound and report here its crystal structure.

Bond lengths and angles in the title molecule (Fig. 1) are found to have normal values (Allen *et al.*, 1987). The furan ring is planar to within ±0.002 (2) Å and the triazole ring is also planar with a maximum deviation of 0.016 (2) Å for atom C1. The triazole and phenyl rings are twisted away from each other by an angle of 16.14 (9)°. The dihedral angle between the furan and triazole rings is 58.51 (11)°. Intramolecular C—H···N hydrogen bonds generate S(5) and S(6) ring motifs (Bernstein *et al.*, 1995).

The crystal structure is stabilized by intermolecular C—H···O and N—H···S hydrogen bonds together with C—H···π interactions involving the phenyl ring. The centrosymmetrically related molecules are linked by N—H···S hydrogen bonds to form a dimeric pair (Fig. 2) which are interlinked via C—H···O hydrogen bonds.

### Experimental

The title Schiff base compound was obtained by refluxing a mixture of 4-amino-5-methyl-2,4-dihydro-3*H*-1,2,4-triazole-3-thione (0.01 mol), furfural (0.01 mol) in ethanol (30 ml) and 2 drops of concentrated H_2_SO_4_ for 3 h. The solid product obtained was collected by filtration, washed with ethanol and dried. Single crystals suitable for X-ray analysis were obtained from acetone-N,N-dimethylformamide (DMF) (1:2) solution by slow evaporation (yield 63%; m.p. 451–453 K). Analysis for C_13_H_10_N_4_SO, found (calculated) in %: C 57.63 (57.77), H 3.62 (3.7), N 20.6 (20.74), S 11.79 (11.85).

### Refinement

The N-bound H atom was located in a difference map and refined with a N-H distance restraint of 0.85 (1) Å. C-bound H atoms were positioned geometrically [C-H = 0.93%A] and refined using a riding model, with *U*_iso_(H) = 1.2*U*_eq_(C).

### Crystal data

**Table:**

C_13_H_10_N_4_OS	*F*_000_ = 1120
*M**_r_* = 270.31	*D*_x_ = 1.464 Mg m^−^^3^
Orthorhombic, *P**b**c**n*	Mo *K*α radiation λ = 0.71073 Å
Hall symbol: -P 2n 2ab	Cell parameters from 5448 reflections
*a* = 27.4006 (6) Å	θ = 2.9–27.9º
*b* = 11.4940 (3) Å	µ = 0.26 mm^−^^1^
*c* = 7.7886 (2) Å	*T* = 100.0 (1) K
*V* = 2452.96 (10) Å^3^	Block, orange
*Z* = 8	0.40 × 0.13 × 0.10 mm

### Data collection

**Table:**

Bruker SMART APEXII CCD area-detector diffractometer	3627 independent reflections
Radiation source: fine-focus sealed tube	2573 reflections with *I* > 2σ(*I*)
Monochromator: graphite	*R*_int_ = 0.072
*T* = 100.0(1) K	θ_max_ = 30.2º
φ and ω scans	θ_min_ = 2.9º
Absorption correction: multi-scan(SADABS; Bruker, 2005)	*h* = −38→38
*T*_min_ = 0.829, *T*_max_ = 0.974	*k* = −16→16
40042 measured reflections	*l* = −10→10

### Refinement

**Table:**

Refinement on *F*^2^	Secondary atom site location: difference Fourier map
Least-squares matrix: full	Hydrogen site location: inferred from neighbouring sites
*R*[*F*^2^ > 2σ(*F*^2^)] = 0.043	H atoms treated by a mixture of independent and constrained refinement
*wR*(*F*^2^) = 0.125	*w* = 1/[σ^2^(*F*_o_^2^) + (0.0649*P*)^2^ + 0.3858*P*] where *P* = (*F*_o_^2^ + 2*F*_c_^2^)/3
*S* = 1.06	(Δ/σ)_max_ = 0.001
3627 reflections	Δρ_max_ = 0.26 e Å^−^^3^
176 parameters	Δρ_min_ = −0.32 e Å^−^^3^
1 restraint	Extinction correction: none
Primary atom site location: structure-invariant direct methods	

### Special details

**Table:**

Experimental. The data was collected with the Oxford Cyrosystem Cobra low-temperature attachment.
Geometry. All e.s.d.'s (except the e.s.d. in the dihedral angle between two l.s. planes) are estimated using the full covariance matrix. The cell e.s.d.'s are taken into account individually in the estimation of e.s.d.'s in distances, angles and torsion angles; correlations between e.s.d.'s in cell parameters are only used when they are defined by crystal symmetry. An approximate (isotropic) treatment of cell e.s.d.'s is used for estimating e.s.d.'s involving l.s. planes.
Refinement. Refinement of *F*^2^ against ALL reflections. The weighted *R*-factor *wR* and goodness of fit *S* are based on *F*^2^, conventional *R*-factors *R* are based on *F*, with *F* set to zero for negative *F*^2^. The threshold expression of *F*^2^ > σ(*F*^2^) is used only for calculating *R*-factors(gt) *etc*. and is not relevant to the choice of reflections for refinement. *R*-factors based on *F*^2^ are statistically about twice as large as those based on *F*, and *R*- factors based on ALL data will be even larger.

### Fractional atomic coordinates and isotropic or equivalent isotropic displacement parameters (Å^2^)

**Table:**

	*x*	*y*	*z*	*U*_iso_*/*U*_eq_	
S1	0.494570 (15)	0.69781 (4)	0.50297 (6)	0.02259 (13)	
O1	0.36669 (4)	1.02453 (10)	0.58350 (18)	0.0258 (3)	
N1	0.44316 (5)	0.52097 (12)	0.6503 (2)	0.0225 (3)	
N2	0.39895 (5)	0.49428 (12)	0.7222 (2)	0.0228 (3)	
N3	0.40473 (5)	0.68093 (11)	0.66047 (19)	0.0190 (3)	
N4	0.38912 (5)	0.79579 (12)	0.6319 (2)	0.0206 (3)	
C1	0.44803 (6)	0.63262 (15)	0.6058 (2)	0.0203 (3)	
C2	0.37554 (6)	0.59359 (14)	0.7269 (2)	0.0204 (4)	
C3	0.32509 (6)	0.60514 (14)	0.7901 (2)	0.0198 (3)	
C4	0.30539 (6)	0.51246 (15)	0.8824 (2)	0.0244 (4)	
H4A	0.3247	0.4484	0.9086	0.029*	
C5	0.25719 (6)	0.51543 (16)	0.9351 (3)	0.0267 (4)	
H5A	0.2444	0.4533	0.9971	0.032*	
C6	0.22781 (6)	0.60973 (16)	0.8967 (2)	0.0259 (4)	
H6A	0.1954	0.6111	0.9320	0.031*	
C7	0.24716 (6)	0.70213 (16)	0.8051 (3)	0.0255 (4)	
H7A	0.2275	0.7656	0.7783	0.031*	
C8	0.29558 (6)	0.70074 (15)	0.7530 (2)	0.0229 (4)	
H8A	0.3084	0.7637	0.6931	0.028*	
C9	0.41795 (6)	0.87250 (15)	0.6957 (2)	0.0212 (4)	
H9A	0.4458	0.8494	0.7548	0.025*	
C10	0.40718 (6)	0.99364 (15)	0.6757 (2)	0.0214 (4)	
C11	0.42966 (7)	1.08948 (16)	0.7401 (3)	0.0268 (4)	
H11A	0.4578	1.0913	0.8067	0.032*	
C12	0.40148 (7)	1.18664 (15)	0.6854 (3)	0.0282 (4)	
H12A	0.4075	1.2646	0.7095	0.034*	
C13	0.36459 (7)	1.14332 (15)	0.5921 (3)	0.0289 (4)	
H13A	0.3406	1.1883	0.5399	0.035*	
H1N1	0.4628 (6)	0.4674 (14)	0.619 (3)	0.034 (6)*	

### Atomic displacement parameters (Å^2^)

**Table:**

	*U*^11^	*U*^22^	*U*^33^	*U*^12^	*U*^13^	*U*^23^
S1	0.0170 (2)	0.0210 (2)	0.0298 (3)	0.00050 (15)	0.00408 (17)	0.00147 (18)
O1	0.0210 (6)	0.0219 (6)	0.0344 (8)	0.0019 (5)	−0.0013 (5)	0.0001 (6)
N1	0.0169 (7)	0.0191 (7)	0.0316 (9)	0.0034 (5)	0.0022 (6)	0.0014 (6)
N2	0.0161 (7)	0.0206 (7)	0.0317 (9)	0.0010 (5)	0.0025 (6)	0.0013 (6)
N3	0.0153 (6)	0.0172 (7)	0.0244 (8)	0.0004 (5)	−0.0002 (6)	0.0008 (6)
N4	0.0185 (6)	0.0173 (7)	0.0260 (8)	0.0016 (5)	0.0010 (6)	0.0015 (6)
C1	0.0170 (7)	0.0208 (8)	0.0232 (9)	0.0014 (6)	−0.0020 (6)	−0.0018 (7)
C2	0.0182 (8)	0.0182 (8)	0.0247 (9)	−0.0013 (6)	−0.0014 (7)	0.0010 (7)
C3	0.0167 (7)	0.0215 (8)	0.0212 (8)	−0.0018 (6)	−0.0011 (6)	−0.0011 (7)
C4	0.0214 (8)	0.0256 (9)	0.0262 (9)	0.0001 (7)	0.0001 (7)	0.0040 (7)
C5	0.0240 (8)	0.0306 (10)	0.0255 (9)	−0.0038 (7)	0.0025 (7)	0.0058 (8)
C6	0.0199 (8)	0.0320 (10)	0.0259 (9)	−0.0012 (7)	0.0031 (7)	−0.0031 (8)
C7	0.0180 (8)	0.0231 (9)	0.0353 (10)	0.0026 (7)	0.0003 (7)	−0.0044 (8)
C8	0.0206 (8)	0.0204 (8)	0.0279 (10)	−0.0013 (6)	0.0008 (7)	0.0008 (7)
C9	0.0161 (7)	0.0240 (8)	0.0236 (9)	0.0006 (6)	0.0024 (7)	0.0003 (7)
C10	0.0169 (7)	0.0235 (9)	0.0238 (9)	−0.0008 (6)	0.0028 (7)	−0.0001 (7)
C11	0.0214 (8)	0.0254 (9)	0.0334 (10)	−0.0042 (7)	0.0033 (7)	−0.0027 (8)
C12	0.0280 (9)	0.0199 (9)	0.0366 (11)	−0.0031 (7)	0.0106 (8)	−0.0019 (8)
C13	0.0280 (9)	0.0216 (9)	0.0372 (11)	0.0054 (7)	0.0072 (8)	0.0050 (8)

### Geometric parameters (Å, °)

**Table:**

S1—C1	1.6820 (17)	C5—C6	1.383 (3)
O1—C13	1.368 (2)	C5—H5A	0.93
O1—C10	1.368 (2)	C6—C7	1.385 (3)
N1—C1	1.336 (2)	C6—H6A	0.93
N1—N2	1.369 (2)	C7—C8	1.388 (2)
N1—H1N1	0.853 (9)	C7—H7A	0.93
N2—C2	1.310 (2)	C8—H8A	0.93
N3—C1	1.377 (2)	C9—C10	1.432 (2)
N3—C2	1.384 (2)	C9—H9A	0.93
N3—N4	1.4054 (18)	C10—C11	1.358 (2)
N4—C9	1.284 (2)	C11—C12	1.423 (3)
C2—C3	1.474 (2)	C11—H11A	0.93
C3—C4	1.394 (2)	C12—C13	1.341 (3)
C3—C8	1.395 (2)	C12—H12A	0.93
C4—C5	1.384 (2)	C13—H13A	0.93
C4—H4A	0.93		
			
C13—O1—C10	105.50 (14)	C5—C6—C7	119.33 (16)
C1—N1—N2	114.19 (14)	C5—C6—H6A	120.3
C1—N1—H1N1	123.8 (15)	C7—C6—H6A	120.3
N2—N1—H1N1	120.9 (15)	C6—C7—C8	120.52 (17)
C2—N2—N1	104.44 (13)	C6—C7—H7A	119.7
C1—N3—C2	108.72 (13)	C8—C7—H7A	119.7
C1—N3—N4	126.29 (13)	C7—C8—C3	120.18 (16)
C2—N3—N4	124.37 (13)	C7—C8—H8A	119.9
C9—N4—N3	113.36 (14)	C3—C8—H8A	119.9
N1—C1—N3	102.77 (14)	N4—C9—C10	119.92 (16)
N1—C1—S1	128.84 (13)	N4—C9—H9A	120.0
N3—C1—S1	128.36 (13)	C10—C9—H9A	120.0
N2—C2—N3	109.79 (14)	C11—C10—O1	110.56 (15)
N2—C2—C3	123.18 (15)	C11—C10—C9	130.92 (17)
N3—C2—C3	127.01 (15)	O1—C10—C9	118.46 (15)
C4—C3—C8	119.00 (15)	C10—C11—C12	106.24 (17)
C4—C3—C2	117.83 (15)	C10—C11—H11A	126.9
C8—C3—C2	123.05 (15)	C12—C11—H11A	126.9
C5—C4—C3	120.26 (16)	C13—C12—C11	106.25 (16)
C5—C4—H4A	119.9	C13—C12—H12A	126.9
C3—C4—H4A	119.9	C11—C12—H12A	126.9
C6—C5—C4	120.71 (17)	C12—C13—O1	111.45 (16)
C6—C5—H5A	119.6	C12—C13—H13A	124.3
C4—C5—H5A	119.6	O1—C13—H13A	124.3
			
C1—N1—N2—C2	−1.5 (2)	C8—C3—C4—C5	0.4 (3)
C1—N3—N4—C9	−60.1 (2)	C2—C3—C4—C5	−175.78 (17)
C2—N3—N4—C9	129.83 (18)	C3—C4—C5—C6	0.3 (3)
N2—N1—C1—N3	2.8 (2)	C4—C5—C6—C7	−0.3 (3)
N2—N1—C1—S1	−175.45 (13)	C5—C6—C7—C8	−0.3 (3)
C2—N3—C1—N1	−2.91 (19)	C6—C7—C8—C3	1.0 (3)
N4—N3—C1—N1	−174.24 (15)	C4—C3—C8—C7	−1.0 (3)
C2—N3—C1—S1	175.32 (14)	C2—C3—C8—C7	174.91 (17)
N4—N3—C1—S1	4.0 (3)	N3—N4—C9—C10	179.42 (14)
N1—N2—C2—N3	−0.50 (19)	C13—O1—C10—C11	0.1 (2)
N1—N2—C2—C3	177.88 (17)	C13—O1—C10—C9	177.54 (16)
C1—N3—C2—N2	2.2 (2)	N4—C9—C10—C11	174.88 (19)
N4—N3—C2—N2	173.76 (15)	N4—C9—C10—O1	−2.0 (3)
C1—N3—C2—C3	−176.07 (17)	O1—C10—C11—C12	0.1 (2)
N4—N3—C2—C3	−4.5 (3)	C9—C10—C11—C12	−176.88 (18)
N2—C2—C3—C4	14.2 (3)	C10—C11—C12—C13	−0.3 (2)
N3—C2—C3—C4	−167.70 (17)	C11—C12—C13—O1	0.4 (2)
N2—C2—C3—C8	−161.76 (17)	C10—O1—C13—C12	−0.3 (2)
N3—C2—C3—C8	16.3 (3)		

### Hydrogen-bond geometry (Å, °)

**Table:**

*D*—H···*A*	*D*—H	H···*A*	*D*···*A*	*D*—H···*A*
N1—H1N1···S1^i^	0.85 (2)	2.42 (2)	3.265 (2)	169 (2)
C4—H4A···N2	0.93	2.55	2.859 (2)	100
C6—H6A···O1^ii^	0.93	2.59	3.347 (2)	139
C8—H8A···N4	0.93	2.29	2.942 (2)	126
C5—H5A···Cg1^iii^	0.93	2.92	3.522 (2)	123

Symmetry codes: (i) −*x*+1, −*y*+1, −*z*+1; (ii) −*x*+1/2, −*y*+3/2, *z*+1/2; (iii) −*x*−1/2, *y*+1/2, *z*.
